# Altered Neural Connectivity in Females, But Not Males with Autism: Preliminary Evidence for the Female Protective Effect from a Quality‐Controlled Diffusion Tensor Imaging Study

**DOI:** 10.1002/aur.2180

**Published:** 2019-07-26

**Authors:** Jiedi Lei, Emma Lecarie, Jane Jurayj, Sarah Boland, Denis G. Sukhodolsky, Pamela Ventola, Kevin A. Pelphrey, Roger J. Jou

**Affiliations:** ^1^ Yale Child Study Center Yale University School of Medicine New Haven Connecticut; ^2^ Centre for Applied Autism Research, Psychology Department University of Bath Bath UK; ^3^ Department of Psychology, Arizona State University Tempe Arizona; ^4^ School of Medicine University of Virginia Charlottesville Virginia

**Keywords:** autism spectrum disorder, diffusion tensor imaging, anisotropy, sex characteristics, motion, female

## Abstract

Previous studies using diffusion tensor imaging (DTI) to investigate white matter (WM) structural connectivity have suggested widespread, although inconsistent WM alterations in autism spectrum disorder (ASD), such as greater reductions in fractional anisotropy (FA). However, findings may lack generalizability because: (a) most have focused solely on the ASD male brain phenotype, and not sex‐differences in WM integrity; (b) many lack stringent and transparent data quality control such as controlling for head motion in analysis. This study addressed both issues by using Tract‐Based Spatial Statistics (TBSS) to separately compare WM differences in 81 ASD (56 male, 25 female; 4–21 years old) and 39 typically developing (TD; 23 males, 16 females; 5–18 years old) children and young people, carefully group‐matched on sex, age, cognitive abilities, and head motion. ASD males and females were also matched on autism symptom severity. Two independent‐raters completed a multistep scan quality assurance to remove images that were significantly distorted by motion artifacts before analysis. ASD females exhibited significant widespread reductions in FA compared to TD females, suggesting altered WM integrity. In contrast, no significant localized or widespread WM differences were found between ASD and TD males. This study highlights the importance of data quality control in DTI, and outlines important sex‐differences in WM alterations in ASD females. Future studies can explore the extent to which neural structural differences might underlie sex‐differences in ASD behavioral phenotype, and guide clinical interventions to be tailored toward the unique needs of ASD females and males. ***Autism Res** 2019, 12: 1472–1483*. © 2019 The Authors. Autism Research published by International Society for Autism Research published by Wiley Periodicals, Inc.

**Lay Summary:**

Previous Diffusion Tensor Imaging (DTI) studies have found atypical brain structural connectivity in males with autism, although findings are inconclusive in females with autism. To investigate potential sex‐differences, we studied males and females with and without autism who showed a similar level of head movement during their brain scan. We found that females with autism had widespread atypical neural connectivity than females without autism, although not in males, highlighting sex‐differences.

## Introduction

Diffusion tensor imaging (DTI) along with other structural and functional imaging techniques, have increasingly been used to provide evidence that the neural phenotype of autism spectrum disorder (ASD) includes alterations in structural connectivity. DTI is a sensitive Magnetic Resonance Imaging (MRI) technique used to detect differences in white matter (WM) architecture by analyzing subtle changes in how water molecules diffuse in different areas of the brain. By modeling bundles of axonal tracts, DTI can be used to infer the nature of physical connections between brain regions at the macroscopic level, and to highlight altered connectivity in individuals with ASD [Weinstein et al., 2011]. Despite prolific research in this area, neuroimaging literature remains inconsistent, due to variability across scanning protocols, image processing, analysis methods, and diagnostic heterogeneity [Jou et al., 2011].

Starting from the first study in 2004 [Barnea‐Goraly et al., 2004], many DTI studies have subsequently demonstrated alterations in fractional anisotropy (FA), a widely accepted measure of structural integrity of WM (i.e., axonal tracts) in the brains of people with ASD [Cheung et al., 2009; Groen, Buitelaar, van der Gaag, & Zwiers, 2011; Shukla, Keehn, Lincoln, & Müller, 2010]. FA is the most common parameter of water diffusion anisotropy and is influenced by nerve fiber density, axonal diameter, and myelination in WM. FA values reflect the directional variation in diffusion [Basser, Mattiello, & LeBihan, 1994], with lower FA values suggesting diffusion across all directions (lower structural integrity) and higher FA values reflecting diffusion parallel to the WM tract (higher structural integrity). Mean diffusivity (MD) represents average diffusion regardless of direction with higher values generally interpreted as lower structural integrity (opposite of FA).

Although previous findings are somewhat heterogeneous, DTI studies in autism have generally demonstrated decreased FA and increased MD in individuals with ASD, and have identified the corpus collosum as a major affected WM tract [Aoki, Abe, Nippashi, & Yamasue, 2013; Rane et al., 2015; Weinstein et al., 2011]. Specifically, decreases in FA were found in the corpus callosum and various association fibers such as the superior longitudinal fasciculus (SLF), occipitofrontal fasciculus, uncinate fasciculus (UF), inferior longitudinal fasciculus (ILF), and cingulum (CING); increases in MD were found in the SLF, corpus callosum, and corticospinal tract (CST) [Cheon et al., 2011; Groen et al., 2011; Jou et al., 2011; Shukla et al., 2010; Walker et al., 2012]. In addition to replicating the finding of WM reductions in the corpus callosum, Di, Azeez, Li, Haque, and Biswal [2018] identified WM reductions in the cerebral peduncle in individuals with ASD.

Fewer DTI studies looked at correlations between imaging parameters and behavioral measures of ASD. Some of these studies reported a negative correlation between FA and autism symptom severity [Cheon et al., 2011; Nair, Treiber, Shukla, Shih, & Müller, 2013; Noriuchi et al., 2010; Sundaram et al., 2008], indicating that lower FA values are associated with higher autism symptom severity. More specifically, studies reported negative correlations between FA and social impairment [Nair et al., 2015] and between WM connectivity and severity of restricted and repetitive behaviors [Thomas, Humphreys, Jung, Minshew, & Behrmann, 2011]. However, it should also be noted that not all studies report significant correlation between neuroimaging and behavioral measures [Jou et al., 2011]. Many studies have run these analyses and have not reported significant findings especially after controlling for multiple comparisons.

The variability in scanning/analysis protocols and data quality control methods may limit the generalizability of these past findings. Koldewyn et al. [2014] matched data quality between ASD and typically developing (TD) groups and found that the previously reported widespread between‐group effects disappeared in all tracts except the right ILF. In other words, what appeared to be a reproducible finding of widespread impairments in structural connectivity were not replicated in more recent studies, which stringently controlled for MRI data quality [Koldewyn et al., 2014; Yendiki, Koldewyn, Kakunoori, Kanwisher, & Fischl, 2014]. Inconsistencies in study findings are common in the autism research literature, especially given the known heterogeneity of the population. However, there is also diversity in data processing and analysis methodology across these studies. Particularly in neuroimaging studies in which tissue contrast is based on subtle differences in water diffusion, stringent methodology should include careful matching and data quality control. In addition to poor matching and data quality control, Yendiki et al. [2014] concluded that variability in head motion can result in group differences in DTI measures. These researchers were able to ameliorate this effect by controlling for head motion in their analysis [Yendiki et al., 2014]. These studies suggest that data quality, suboptimal group matching, and head motion create major discrepancies in which specific tracts are identified as being affected in individuals with autism.

In addition to data quality considerations, future research should place an emphasis on studying females and males separately in order to clarify sex specific differences in ASD. Past research has primarily investigated differences between ASD and TD groups, using either male samples or mixed sex samples. Lai, Lombardo, Auyeung, Chakrabarti, and Baron‐Cohen [2015] reviewed sex differences in autism and proposed future research should examine developmental mechanisms of ASD separately in females and in males, given that these mechanisms are likely sex‐specific. Given the inconsistent findings using relatively small mixed sex samples in the previous literature [Beacher et al., 2012; Kirkovski, Enticott, Maller, Rossell, & Fitzgerald, 2015], the differences in the neural phenotypes between males and females with ASD should be considered in conjunction with study designs that supports clear comparisons between males and females.

It is readily believed that there are sex differences in neurotypical brains. Moreover, there are major sex differences in the diagnosis of ASD [Ferri, Abel, & Brodkin, 2018]. Researchers have proposed various explanations to interpret the higher rate of ASD diagnosis in males compared to females. The “Extreme Male Brain” (EMB) theory suggests that sex differences exist in cognitive and affective processes [Baron‐Cohen, 2002]. This cognitive‐based theory posits that males demonstrate greater “systemizing” compared to “empathizing,” while females exhibit the opposite pattern. Baron‐Cohen [2002] explains systemizing to be an inductive process, which involves analyzing the rules of systems. By comparison, female cognition aligns best with the framework of empathizing, suggesting that females are more adept in interpreting affective states, and using their interpretations to understand the social world [Baron‐Cohen, 2002]. The theory suggests that males with ASD may reach a “ceiling,” such that it is more difficult to identify neural markers of ASD in males than it is in females.

Plausible explanations supporting sex differences grounded in neurogenetics, neuroanatomy, and neural connectivity are outlined in a review by Chen, Van Horn, and GENDAAR Research Consortium [2017]. This review proposed that multiple genetic and environmental factors are responsible for producing a “female protective effect.” This female protective effect posits that a greater genetic mutational load is required for the ASD phenotype to manifest in females, than it is needed in their male counterparts. Werling [2016] reviewed multiple female‐specific genetic and biological factors and their potential role in the female protective effect, and noted that in the unaffected population, females scored lower on ASD traits, such that ASD risk factors were less likely to push females into the ASD diagnostic category. Other studies have examined evidence of sex differences in the neuroanatomy and connectivity in ASD has also been reviewed. For example, Beacher et al. [2012] found that the FA in the right and left CING, corpus callosum, and corona radiata is greater in control adult males compared to control adult females, but that the FA does not significantly differ between adult males and females with ASD. Irimia, Torgerson, Jacokes, and Van Horn [2017] employed DTI and MRI techniques to identify neural correlates of the sex‐by‐ASD diagnosis interaction effect. This research concluded that the sex‐by‐ASD diagnosis interaction effect observed in WM connectivity density at the group level was statistically modulated by WM connectivity density in the lateral temporal lobe, medial parietal lobe, and temporo‐parieto‐occipital junction. The greater WM connectivity density differences found between ASD and TD males in these regions suggest greater developmental impairment in a number of brain regions involved in language processing (lateral temporal lobe), executive function and motor planning (medial parietal lobe), and social cognition (temporo‐parieto‐occipital junction) in ASD males [Irimia et al., 2017].

Although currently a major area in inquiry, studies of sex differences in autism are less common in the MRI research literature. Mixed findings have been reported in the few available studies using DTI to investigate sex differences in neural connectivity in ASD. DTI has been used to show increased FA and other diffusion measures (radial and axial diffusivity [AD]) in females with ASD (*n* = 27) compared to TD females (*n* = 29), while observing no such increases in males (*n* = 112) with ASD compared with their neurotypical peers (*n* = 53) in a group of young children aged 3–5 years old [Nordahl et al., 2015]. In comparison, Beacher et al. [2012] observed reduced FA in males with ASD (*n* = 15) compared to TD males (*n* = 15), while detecting no difference between females with ASD (*n* = 13) and TD females (*n* = 15) in a group of adults aged 30–32 years. In addition to inconsistencies in DTI findings that support sex differences in ASD, Kirkovski et al. [2015] found no effect of sex on FA and other diffusivity measures using a sample of 12 males with ASD, 13 females with ASD, 12 TD males, and 12 TD females in a group of adults aged 27–33 years. It is important to note these studies were limited by sample size, and future studies should aim to recruit a greater sample of female ASD participants. Although some of the discrepant findings need to be interpreted within the context of development due to differences in participants' age across studies (i.e., comparing children to adults), the differences observed in Beacher et al. [2012] and Kirkovski et al. [2015] with participants of a similar age range and sample size suggest other factors associated with study methodology beyond age differences may also account for some of the variance across findings. Therefore, the degree of variability among these findings highlights the need for strict methodology, group matching, and stringent data quality control, when using DTI to investigate structural neural phenotypes in ASD.

The present study aims to evaluate sex differences in WM between individuals with ASD and their TD peers, while stringently controlling for data quality and matching for confounding variables known to produce artifact findings and false positive errors. This aligns with the notion of controlling for the effect of head motion highlighted by Koldewyn et al. [2014], in which TD and ASD participants were matched by head motion and samples with much excessive head motion were not included in analyses. The present study builds on previous research because of the data quality method used and the inclusion of behavioral measures in relation to the imaging parameters. The study also challenges previous research that reports significant differences in WM tracts in the ASD group by analyzing males and females as separate groups to capture any evident sex differences.

## Materials and Methods

### *Subjects*

Study participants included 81 children and adolescents with ASD (56 male, 25 female; age 4–21 years), and 39 TD children and adolescents (23 male, 16 female; age 5–18 years). Participant characterization can be seen in Table 1. All participants were recruited as part of a larger project that focused on investigating social cognition differences in autism taken place at the Yale Child Study Center. In the context of the current study, sex is referred to as biological sex that parents reported on the demographic information during recruitment. We did not directly measure gender such as by asking the participants their preferred pronoun or gender identity, and therefore sex in the current study is used strictly in the biological sense as per parental report. All ASD participants had a prior diagnosis, made by a clinical professional, of either Autistic Disorder, Asperger's Disorder, or Pervasive Developmental Disorder—Not Otherwise Specified according to *Diagnostic and Statistical Manual of Mental Disorders (DSM), 4th Edition*; or ASD according to *DSM 5th Edition* [American Psychiatric Association, 2013]. In addition to clinical history, diagnostic information was confirmed upon entering the study by parental information based on Autism Diagnostic Interview‐Revised (ADI‐R; Rutter, LeCouteur, & Lord, 2003), and expert evaluation using the Autism Diagnostic Observation Schedule (Lord et al., 2012). Other than a diagnosis of ASD, parents reported an absence of medical and neurologic disease for all participants with ASD.

**Table 1:** Demographics and Group Characterization

	ASD				TD			
	Female (*n* = 25)		Male (*n* = 56)		Female (*n* = 15)		Male (*n* = 23)	
	Mean (SD)	Range	Mean (SD)	Range	Mean (SD)	Range	Mean (SD)	Range
*Age (years)*	9.30 (4.25)	4–21	9.63 (4.02)	4–18	11.03 (4.24)	4–17	10.91 (4.51)	4–18
*FSIQ*								
GCA^a^	97.20 (21.34)	63–134	97.34 (21.00)	46–158	107.27 (14.97)	85–143	103.00 (18.61)	73–141
Verbal	97.88 (22.10)	62–140	96.55 (22.86)	35–146	110.20 (18.04)	92–165	105.35 (18.07)	73–154
Nonverbal	99.13 (20.71)	60–136	98.36 (18.49)	54–158	101.80 (11.95)	77–121	101.74 (20.66)	69–153
Spatial	96.78 (20.63)	64–137	97.56 (18.96)	55–138	106.13 (17.60)	79–146	99.87 (13.54)	75–118
*SRS raw score*	(*n* = 23)		(*n* = 49)		(*n* = 12)		(*n* = 21)	
Social affect	12.00 (3.46)	6–20	12.04 (3.81)	2–20	4.58 (2.35)	1–8	4.57 (2.73)	0–12
Social cognition	15.74 (4.44)	8–26	16.88 (6.36)	3–31	5.50 (7.48)	0–22	2.90 (3.73)	0–14
Social communication	31.91 (9.32)	17–51	30.86 (10.89)	5–51	8.50 (10.45)	0–34	6.24 (6.34)	0–28
Social motivation	13.78 (4.32)	3–29	13.16 (5.30)	0–24	5.50 (4.96)	0–15	3.48 (2.71)	0–9
Autistic mannerisms	15.30 (4.32)	4–20	17.55 (7.52)	1–34	3.75 (5.12)	0–14	2.62 (3.63)	0–16
Total	88.74 (22.67)	52–144	90.53 (30.15)	18–147	27.83 (28.38)	3–93	19.81 (17.08)	3–77

a Measured with Differential Abilities Scale, second edition (DAS‐II).

Abbreviations: ASD, autism spectrum disorder; FSIQ, full scale IQ; GCA, general conceptual ability; SRS, Social Responsiveness Scale; TD, typically developing.

For the TD group, parents reported that participants did not have any history of neurologic disease, neurologic insult resulting in loss of consciousness, other psychiatric disorders, or history of ASD in first‐ or second‐degree relatives. All participants in both the ASD and TD groups also completed either the *Differential Abilities Scale II*—*Early Years/School Age* (DAS‐II; Elliott, 2007) if younger than 18 years old, or the Wechsler Adult Intelligence Scale (Wechsler, 2008) if above 18 years old to assess their cognitive functioning. In order to reflect the most clinically meaningful IQ scores obtained, only scores from IQ assessments completed within 1 year of the neuroimaging scan date are reported in this study. Only one TD female who was included in the current analysis did not have a valid IQ assessment completed within 1 year of the neuroimaging scan data. Parents also completed the *Social Responsiveness Scale—Second Edition* (SRS‐2; Constantino & Gruber, 2012), a 65‐item questionnaire that primarily assesses social communication competency in children and adolescents, and is taken as a measure of autism symptom severity. SRS‐2 has high concurrent validity with ADI‐R scores (*r* = 0.6–0.79), and has high test re‐test reliability (*r* = 0.88) in clinical populations [Constantino et al., 2003]. To assess whether the ASD and TD groups were matched by age and IQ (full scale IQ [FSIQ]), as well as evaluate differences in ASD symptom severity across groups, we conducted a 2 (sex) × 2 (diagnosis) multivariate analysis of variance (MANOVA), using the Bonferroni method to correct for multiple comparisons.

### *Ethical Approval*

The Human Investigation Committee at Yale University approved this research, and all procedures are conducted in line with the Declaration of Helsinki in 1964 and its later amendments. Written informed consent and assent from all participants and their parent/primary caregiver were obtained prior to their participation in the study. All participation in the study was voluntary, and participants and their family were reminded that they can withdraw from the study at any time without needing to provide a reason for withdrawal.

### *Image Acquisition*

MR imaging were acquired using a 3T Magnetom Tim Trio system (Siemens, Erlangen, Germany). Diffusion‐weighted data were collected with an 8‐channel head coil, using parallel imaging to gain better signal intensity at air‐tissue interfaces. Diffusion imaging parameters include: diffusion directions = 30, B0 = 5, Repetition Time (TR) = 6,200 ms, Echo time (TE) = 85 ms, Field‐of‐View (FOV) = 240 mm × 240 mm, section thickness = 2.5 mm (isotropic), Generalised Autocalibrating Partial Parallel Acquisition (GRAPPA) on, number of slices = 55, average = 3, and total scan time = 11 min. With a standard single‐channel head coil, whole‐brain T1‐weighted MR imaging was performed using a sagittal 1‐mm^3^ magnetization‐prepared rapid acquisition of gradient echo sequence. The pulse sequence parameters were as follows: TR = 2,530 ms, TE = 3.66 ms, TI = 1,100 ms, flip angle = 7°, Number of Excitations (NEX) = 1, number of sections = 176, bandwidth = 181 Hz/pixel, matrix = 256 × 256, FOV = 256 mm × 256 mm, GRAPPA off, and scan time = 8 min. All imaging was performed during a single session.

### *Image Processing and Analyses*

Functional Magnetic Resonance Imaging of the Brain (FMRIB) Software Library (FSL, https://www.fmrib.ox.ac.uk/fsl) was used to conduct data preprocessing and local diffusion modeling. Rather than using the Benner score for data quality control as in Koldewyn et al. [2014], a multistep scan quality assurance involving visual inspection was completed. First, all volumes were inspected by an experienced rater for severe motion and other artifacts. Using pre‐established criteria, a run was excluded if the image quality of six or more directions were compromised due to geometric distortion, magnetic susceptibility effects, and other severe motion or artifacts. A second trained rater independently viewed a randomly selected 10% of all runs, and used the same multistep scan quality assurance to exclude volumes in the selected runs. Inter‐rater reliability for volume exclusion was calculated (intraclass correlation coefficient >0.90). Diffusion‐weighted images were then corrected for eddy current distortion and simple head motion. Next, for each participant, the remaining volumes across all remaining runs after quality assurance were averaged to improve signal intensity‐to‐noise ratio. FSL was then used to generate a mask to separate brain from nonbrain areas. The binary brain mask, averaged diffusion‐weighted data, *b*‐values, and vector information were then inputted into FMRIB Diffusion Toolbox, which fits a diffusion tensor model at each voxel, thus generating a FA map for each subject. Head motion for all participants in terms of absolute (transformation matrix displacement between time point *N* and baseline reference point) and relative (transformation matrix displacement between time point *N* and *N* + 1) displacement, rotation, and translation were calculated. We conducted a 2 (sex) × 2 (diagnosis) MANOVA to assess any potential differences in head motion severity across the groups.

To overcome the shortcomings of voxel‐based statistics of FA images whereby there is often a lack of standard registration algorithm for aligning FA images of multiple subjects, we chose to use Tract‐Based Spatial Statistics (TBSS), which projects individual FA maps onto the alignment‐invariant FA skeleton following automated nonlinear registration. TBSS thus provides a more satisfactory solution for allowing valid conclusions to be drawn from diffusion imaging studies that involve multiple subjects. The following procedure was implemented: (a) conversion of FA data into an appropriate format, (b) application of nonlinear registration so all FA images are in Montreal Neurological Institute (MNI) space; (c) creation of a mean FA image separately for males and females; (d) skeletonization of mean FA image by using FA threshold of 0.3; (e) projection of all subjects' FA data onto the mean FA skeleton (created within each sex); and (f) submission of the 4D‐projected FA data for statistical testing. Given the current study had a main focus on identifying phenotypic sex differences between ASD and TD participants, we then performed voxel‐wise analysis between ASD and TD for males and females using the multisubject diffusion data. Areas of significant differences were computed and displayed as *P* value image, where *P* < 0.05, using randomize and threshold‐free cluster enhancement (TFCE) to correct for multiple comparisons across space. Finally, although not the primary focus of the current study, we also completed exploratory analysis using the same TBSS and TFCE procedure as above to examine potential sex differences between ASD and TD for males and females in AD, radial diffusivity (RD), and MD.

We used the Johns Hopkins University (JHU) WM Tractography Atlas included in the FSL software package to identify the affected WM structures with greater precision. The JHU WM Tractography Atlas helped to identify for both potentially affected fiber tracts (i.e., identifying the tracts upon which voxels of significant difference might lie), and additionally allotted for more precise characterization of potential pathology by quantifying affected voxels based on their tract labels. Detailed MNI coordinates of all affected voxels were captured and intersected with the JHU WM Tractography Atlas. Aside from the voxels that were numbered according to the atlas (i.e., no label), the remaining voxels were each assigned a certain percentage according to tract labels from 1 to 21, which consists of association, commissural, and project tracts. The tract with the highest percentage of representation was chosen as the designated label for that particular voxel. For each participant, affected voxels were grouped by label with their corresponding FA, and total voxel counts were generated for each fiber tract. The mean FA, SD, effect size (Cohen's *d*), and asymmetry index were computed for each fiber tract. Finally, exploratory Pearson correlation analyses were performed between mean FA data of the most severely affected fiber tracts and SRS scores. Bonferroni correction was used to control for multiple comparisons. All statistical analyses were conducted using Version 21.0 [IBM Corp, 2012].

## Results

### *Demographics*

Using ANOVA, no significant main effect of group was observed for age (*F*(1, 115) = 3.10; partial eta squared = 0.026; *P* = 0.081) or FSIQ (*F*(1, 115) = 3.69; partial eta squared = 0.031; *P* = 0.057). For the SRS‐2, multivariate analysis found a significant main effect of group for the SRS total score (*F*(1, 101) = 128.96; partial eta squared = 0.56; *P* < 0.001), as well as all subscale scores including social affect (*F*(1, 101) = 98.18; partial eta squared = 0.49; *P* < 0.001); social cognition (*F*(1, 101) = 92.97; partial eta squared = 0.48; *P* < 0.001); social communication (*F*(1, 101) = 124.47; partial eta squared = 0.55; *P* < 0.001); social motivation (*F*(1, 101) = 63.05; partial eta squared = 0.38; *P* < 0.001); and autistic mannerisms (*F*(1, 101) = 98.81; partial eta squared = 0.50; *P* < 0.001). Post hoc analyses revealed that the ASD group scored higher than the TD group on SRS‐2 total and all subscale scores (*P* < 0.001), indicative of greater autism symptom severity. No main effect of sex was observed for age (*F*(1, 115) = 0.02; partial eta squared < 0.001; *P* = 0.90), FSIQ (*F*(1, 115) = 0.25; partial eta squared = 0.002; *P* = 0.62), or SRS‐2 total or any subscale scores (*F*(1, 101) = 0.00–1.36; partial eta squared <0.001–0.01; *P* = 0.25–0.99). No group by gender interactions was observed.

### *Head Motion*

Using MANOVA, no significant main effect of group (Pillai's trace = 0.04; *F*(8, 108) = 0.77; partial eta squared = 0.04; *P* = 0.77) or sex (Pillai's trace = 0.07; *F*(8, 108) = 1.10; partial eta squared = 0.08; *P* = 0.37), or any group × sex interactions (Pillai's trace = 0.03; *F*(8, 108) = 0.46; partial eta squared = 0.03; *P* = 0.88) emerged for any motion parameters measured (Table 2).

**Table 2:** Head Motion Characterization

	ASD				TD			
	Female (*n* = 25)		Male (*n* = 56)		Female (*n* = 15)		Male (*n* = 23)	
	Mean (SD)	Range	Mean (SD)	Range	Mean (SD)	Range	Mean (SD)	Range
*Displacement (mm)*								
Absolute	3.46 (0.91)	1.49–7.82	3.18 (1.45)	1.54–8.47	3.04 (1.12)	1.62–5.26	2.83 (1.71)	1.42–8.35
Relative	1.27 (0.41)	0.73–2.22	1.05 (0.32)	0.60–1.95	1.21 (0.27)	0.61–1.65	1.03 (0.33)	0.49–2.01
*Rotation (mm)*								
*X*	0.00 (0.02)	−0.04 to 0.03	0.00 (0.02)	−0.09 to 0.05	0.01 (0.01)	−0.02 to 0.03	0.00 (0.03)	−0.08 to 0.08
*Y*	0.00 (0.01)	−0.03 to 0.02	0.00 (0.01)	−0.05 to 0.03	0.00 (0.01)	−0.02 to 0.01	0.00 (0.01)	−0.02 to 0.02
*Z*	0.00 (0.01)	−0.02 to 0.01	0.00 (0.01)	−0.05 to 0.02	0.00 (0.01)	−0.01 to 0.03	0.00 (0.01)	−0.03 to 0.01
*Translation (mm)*								
*X*	−0.05 (0.33)	−1.02 to 0.54	−0.12 (0.39)	−1.77 to 0.79	−0.04 (0.39)	−0.87 to 0.9	−0.08 (0.30)	−1.06 to 0.32
*Y*	0.91 (0.47)	−0.07 to 1.85	0.71 (0.52)	−1.34 to 1.98	0.88 (0.49)	0.29–2.03	0.79 (0.37)	0.17–1.92
*Z*	1.32 (1.24)	−0.72 to 4.41	1.27(1.17)	−0.93 to 4.46	1.04 (0.66)	−0.10 to 2.19	0.80 (0.59)	−0.49 to 1.81
*% slices excluded*	13.75 (14.82)	0–54.29	10.71 (10.82)	0–42.86	18.06 (12.52)	1.43–41.90	13.98 (16.47)	0–61.43

*Note*. No statistical significances were observed across diagnostic groups or gender.

Abbreviations: ASD, autism spectrum disorder; SD, standard deviation; TD, typically developing.

### *TBSS*

**Figure 1:**
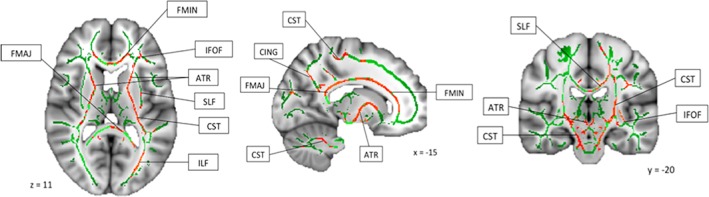
Widespread areas of reduced fractional anisotropy in autism spectrum disorder group compared with typically developing group. Abbreviations: ATR, anterior thalamic radiation; CING, cingulum; CST, corticospinal tract; FMAJ, forceps major; FMIN, forceps minor; IFOF, inferior fronto‐occipital fasciculus; ILF, inferior longitudinal fasciculus; SLF, superior longitudinal fasciculus; UNF, uncinate fasciculus.

The main results of this study are summarized in Table 3 and Figure 1. For males, we did not observe any significant differences in FA across any fiber tracts when comparing the ASD group to the TD group. In contrast, for females, the ASD group showed significant reductions in FA across association, projection, and commissural fibers when compared to the TD group. Bilateral FA reductions were found in association tracts including the CING, inferior fronto‐occipital fasciculus (IFOF), ILF, SLF, and UF. Affected projection tracts included bilateral FA reductions in the anterior thalamic radiation (ATR) and the CST. Affected commissural tracts included bilateral FA reductions in both the forceps major (FMAJ) and forceps minor (FMIN). Figure 1 shows the widespread distribution of the affected long‐range fibers in females where the TD group showed increased FA compared to the ASD group.

**Table 3:** Characteristics of Affected Long‐Range Fiber Tract by Type Using Tract‐Based Spatial Statistics (TBSS) in Females

				Mean fractional anisotropy (SD)		
Affected long‐range tract	Total number of voxels (FA skeleton)	Number (%) of voxels affected	% Asymmetry^a^ (left vs. right) voxels affected	ASD (*n* = 25)	TD (*n* = 16)	Cohen's *d*
*Association tracts*						
Left CING	2,378	527 (22.16)	4.63	0.46152 (0.02614)	0.48998 (0.03317)	0.95
Right CING	1,745	336 (19.26)		0.47130 (0.02113)	0.49520 (0.02518)	1.03
Left IFOF	3,608	1,195 (33.12)	8.58	0.43450 (0.02626)	0.46620 (0.01997)	1.36
Right IFOF	5,683	398 (7.00)		0.45285 (0.03708)	0.48516 (0.03674)	0.88
Left ILF	3,158	543 (17.19)	9.06	0.45816 (0.03315)	0.49328 (0.02122)	1.26
Right ILF	2,590	22 (0.85)		0.45408 (0.05137)	0.48973 (0.04963)	0.71
Left SLF	7,048	1,034 (14.67)	6.88	0.41778 (0.02351)	0.44296 (0.02610)	1.01
Right SLF	6,931	72 (1.04)		0.46865 (0.02663)	0.49628 (0.03802)	0.84
Left UNF	1,061	165 (15.55)	49.24	0.36560 (0.02333)	0.39665 (0.03010)	1.15
Right UNF	734	4 (0.54)		0.42627 (0.03314)	0.44620 (0.03857)	0.55
*Projection tracts*						
Left ATR	5,156	1,956 (37.94)	6.72	0.44458 (0.02366)	0.47308 (0.02788)	1.10
Right ATR	4,231	1,325 (31.32)		0.46010 (0.02427)	0.48957 (0.03166)	1.04
Left CST	4,961	1,602 (32.29)	3.13	0.52994 (0.02414)	0.55624 (0.02501)	1.07
Right CST	4,939	1,292 (26.16)		0.53947 (0.02680)	0.56752 (0.02766)	1.03
*Commissural tracts*						
FMAJ	3,795	1,268 (33.41)	NA	0.57227 (0.03192)	0.60807 (0.02923)	1.17
FMIN	5,035	1,618 (32.14)	NA	0.62022 (0.02273)	0.64723 (0.02141)	1.22

a Asymmetry for number of voxels affected is calculated by the dividing the difference in number of voxels affected in each tract between the left and right hemisphere by the total number of voxels in that tract as per FA skeleton.

Abbreviations: ASD, autism spectrum disorder; ATR, anterior thalamic radiation; CING, cingulum; CST, corticospinal tract; FMAJ, forceps major; FMIN, forceps minor; IFOF, inferior fronto‐occipital fasciculus; ILF, inferior longitudinal fasciculus; SLF, superior longitudinal fasciculus; TD, typically developing; UNF, uncinate fasciculus.

As shown in Table 3, not all WM fiber tracts were equally affected, and significant variation was not only observed across fiber tracts, but also across hemispheres. Effect sizes are shown in Cohen's *d* (0.2 = small, 0.5 = medium, 0.8 = large), used here as a metric for relative comparison of the effects observed across different tracts, rather than taken as an absolute measure of effect size for each tract. Due to potential inflation of the effect sizes during the TBSS analyses process, and subsequent exaggeration of results, the effect sizes are not interpreted by their absolute values. Therefore, caution should be taken and the effect sizes reported here are used for relative comparison across different fiber tracts only. Although all fiber tracts were affected, we consistently observed more widespread reduced FA in the left hemisphere in females with ASD when compared to TD females. This observation is best captured in the asymmetry index, calculated as the difference found in each tract between the left and right hemisphere, divided by the total number of voxels for that tract in both left and right hemisphere in the FA skeleton. The fiber tracts with the greatest number of voxels affected included all the projection tracts (bilateral ATR and bilateral CST), commissural tracts (FMAJ and FMIN), and also the left IFOF and left SLF. We did not observe any significant increases in FA across any fiber tracts in females with ASD when compared to TD females. No statistically significant differences were observed in AD, RD, or MD across any fiber tracts between either male or female ASD versus TD comparison.

### *Autism Symptom Severity and WM Differences*

Finally, we conducted exploratory Pearson's correlation between autism symptom severity, as measured by the subscales and total raw scores from SRS‐2, and mean FA data for females in both ASD and TD group, using the Bonferroni method to correct for multiple comparisons. We did not observe any significant correlations for either group across any of the fiber tracts or with any subscale or total scores from SRS‐2, suggesting no significant correlation between WM differences and autism symptom severity in the ASD group, or autistic traits in the TD group (Table 4).

**Table 4:** Pearson's Correlation Coefficient (*r*; *P* value in brackets) Between Autism Symptom Severity (Social Responsiveness Scale‐2 Raw Scores) and White Matter Differences as Identified by Fractional Anisotropy in Females

	ASD (*n* = 23)						TD (*n* = 12)					
	Soc Affect	Soc Cog	Soc Comm	Soc Mot	Mannerisms	Total	Soc Affect	Soc Cog	Soc Comm	Soc Mot	Mannerisms	Total
*Association tracts*												
Left CING	0.06 (0.80)	0.23 (0.29)	0.05 (0.81)	0.35 (0.10)	0.10 (0.65)	0.19 (0.38)	−0.01 (0.99)	−0.12 (0.72)	−0.24 (0.49)	−0.26 (0.45)	−0.20 (0.55)	−0.20 (0.56)
Right CING	0.27 (0.21)	0.33 (0.13)	0.26 (0.24)	0.50 (0.02)	0.33 (0.12)	0.41 (0.05)	0.12 (0.73)	0.11 (0.76)	−0.11 (0.77)	0.05 (0.89)	0.10 (0.76)	0.03 (0.94)
Left IFOF	−0.06 (0.79)	0.07 (0.74)	0.01 (0.95)	0.29 (0.18)	−0.02 (0.93)	0.09 (0.69)	0.25 (0.47)	−0.01 (0.99)	−0.14 (0.68)	−0.25 (0.46)	−0.05 (0.88)	−0.09 (0.80)
Right IFOF	0.03 (0.91)	0.17 (0.44)	0.16 (0.47)	0.19 (0.38)	0.05 (0.81)	0.17 (0.45)	0.11 (0.76)	0.23 (0.49)	0.13 (0.70)	0.12 (0.72)	0.09 (0.78)	0.16 (0.65)
Left ILF	−0.13 (0.55)	0.03 (0.91)	−0.06 (0.77)	0.24 (0.27)	−0.16 (0.47)	−0.01 (0.98)	0.27 (0.42)	−0.01 (0.98)	−0.05 (0.88)	−0.02 (0.96)	0.09 (0.79)	0.02 (0.96)
Right ILF	0.22 (0.32)	0.32 (0.14)	0.32 (0.14)	0.19 (0.38)	0.04 (0.85)	0.29 (0.18)	0.28 (0.41)	0.26 (0.44)	0.27 (0.43)	0.26 (0.45)	0.17 (0.62)	0.27 (0.43)
Left SLF	−0.10 (0.65)	−0.02 (0.94)	−0.15 (0.51)	0.25 (0.25)	0.03 (0.88)	−0.00 (0.99)	0.29 (0.40)	0.04 (0.90)	−0.02 (0.96)	−0.01 (0.97)	0.02 (0.97)	0.03 (0.93)
Right SLF	0.04 (0.87)	0.12 (0.58)	0.25 (0.25)	0.42 (0.04)	0.19 (0.38)	0.29 (0.19)	0.43 (0.19)	0.20 (0.55)	0.01 (0.97)	0.03 (0.92)	0.20 (0.57)	0.14 (0.69)
Left UNF	0.04 (0.87)	−0.10 (0.66)	−0.07 (0.77)	−0.04 (0.87)	−0.32 (0.13)	−0.11 (0.61)	0.13 (0.70)	−0.19 (0.57)	−0.25 (0.46)	−0.34 (0.30)	−0.25 (0.45)	−0.24 (0.49)
Right UNF	0.18 (0.41)	0.02 (0.91)	0.07 (0.76)	0.24 (0.27)	0.15 (0.51)	0.16 (0.47)	0.29 (0.40)	−0.17 (0.62)	0.05 (0.87)	−0.17 (0.62)	−0.13 (72)	−0.05 (0.88)
*Projection tracts*												
Left ATR	0.06 (0.78)	−0.02 (0.94)	0.03 (0.91)	0.31 (0.15)	−0.02 (0.94)	0.10 (0.65)	0.24 (0.48)	0.11 (0.76)	−0.04 (0.90)	−0.12 (0.72)	0.03 (0.93)	0.02 (0.96)
Right ATR	0.13 (0.55)	0.05 (0.83)	0.12 (0.58)	0.32 (0.13)	0.02 (0.92)	0.17 (0.43)	0.26 (0.45)	0.18 (0.59)	0.03 (0.94)	−0.05 (0.88)	0.06 (0.85)	0.08 (0.81)
Left CST	0.03 (0.89)	0.01 (0.97)	−0.07 (0.75)	0.29 (0.18)	0.00 (0.99)	0.06 (0.79)	0.19 (0.58)	0.04 (0.91)	−0.08 (0.82)	−0.12 (0.72)	−0.06 (0.86)	−0.04 (0.92)
Right CST	0.05 (0.82)	0.13 (0.54)	0.11 (0.60)	0.36 (0.09)	0.02 (0.92)	0.19 (0.39)	0.22 (0.51)	0.09 (0.79)	−0.05 (0.89)	−0.15 (0.66)	−0.02 (0.96)	−0.00 (0.99)
*Commissural tracts*												
FMAJ	0.06 (0.78)	0.23 (0.29)	0.10 (0.67)	0.32 (0.14)	−0.04 (0.85)	0.17 (0.43)	0.04 (0.92)	−0.09 (0.80)	−0.31 (0.36)	−0.17 (0.62)	−0.15 (0.66)	−0.19 (0.58)
FMIN	−0.02 (0.91)	−0.04 (0.86)	−0.01 (0.98)	0.17 (0.44)	0.09 (0.69)	0.05 (0.82)	0.31 (0.36)	−0.00 (0.99)	−0.03 (0.93)	−0.12 (0.74)	−0.01 (0.97)	−0.01 (0.98)

*Note*. Bonferroni corrected alpha = 0.01.

Abbreviations: ASD, autism spectrum disorder; ATR, anterior thalamic radiation; CING, cingulum; CST, corticospinal tract; FMAJ, forceps major; FMIN, forceps minor; IFOF, inferior fronto‐occipital fasciculus; ILF, inferior longitudinal fasciculus; SLF, superior longitudinal fasciculus; Soc Affect, social affect; Soc Cog, social cognition; Soc Comm, social communication; Soc Mot, social motivation; TD, typically developing; UNF, uncinate fasciculus.

## Discussion

The current report addresses controversy around the role data quality plays in DTI findings in autism: consistently inconsistent yet widespread. The working hypothesis is that these inconsistencies can be attributed to heterogeneity in autism phenotype and research methodology. In this study, we stringently controlled research methodology to produce findings that more accurately reflect the neural phenotype of autism. In addition, this study includes separate analyses of females and males to evaluate sex differences in a way that was not possible in previous male only, or mixed samples with few females.

We hypothesized that the results of this study would approximate those reported by Koldewyn et al. [2014] and that girls with autism would exhibit similar neural phenotype as boys. We expected this study to replicate the localized finding of altered connectivity in the left ILF. Koldewyn et al. [2014] used a mixed sample including boys and girls in both ASD and TD groups therefore strict replication was unlikely. However, the current study supported neither localized nor widespread WM alterations in ASD boys. By contrast to null findings in ASD boys, ASD girls exhibited significant reductions in FA when compared to TD girls. This finding in girls was not localized to a single fiber tract but scattered across the range of association commissure and projection tracts. There were no areas where FA was significantly increased in ASD girls. The finding is similar to those reported in boys and mixed samples; however, in our study the left hemisphere was disproportionately affected. Since autism is characterized by challenges in social communication, the dominant effects in the left hemisphere align with classic neurologic teaching of the human brain function. The left side of the brain is well‐known for its role in language and communication [Balsamo et al., 2002; Vigneau et al., 2006]; therefore, these alterations may correlate with behavioral measures dependent on language. Nagae et al. [2012] identified increased MD in the ASD cohort of the left hemisphere SLF, and found that this alteration was especially pronounced in a subgroup of ASD individuals with a language impairment. Cauda et al. [2014] found a more pronounced negative concordance between WM and GM alterations in the right hemisphere SLF of individuals with ASD compared to TD controls and interpreted this right hemisphere lateralization as it relates to visuospatial processing, semantics, and prosody of language. Although correlation analyses in the present study did not reveal significant findings, the disproportion effect in the left hemisphere as it relates to language and communication should further be investigated in females.

Widespread WM alterations detected only in ASD girls despite comparable SRS‐2 scores between sexes, suggest that comparable symptoms of autism require substantial structural aberrations in girls with autism. These results align with the notion that females require a greater genetic load to manifest the behavioral phenotype of autism, and therefore provide preliminary support for the female protective effect [Robinson, Lichtenstein, Anckarsäter, Happé, & Ronald, 2013]. Through this framework, we would expect that females in our sample demonstrate a greater etiological load, and therefore display alterations in neural connectivity that the males in our sample do not.

In addition, our results can be understood through the cognitive level framework of Baron‐Cohen [2002]'s EMB theory. Baron‐Cohen [2002]'s hypothesis that ASD is in fact an exaggeration of the typical male systemizing to empathizing quotient, implies that males require a less dramatic shift in this discrepancy between systemizing and empathizing to display the ASD phenotype than females. As a result of the magnitude of the difference between TD female brain, and the EMB that is characteristic of ASD, it may be easier to detect neural differences between ASD females and TD females, than it is to detect these same between group differences in males. This hypothesis is consistent with our finding of significant differences in FA between ASD females and TD females, but no comparable effect between ASD males and TD males.

There are four key limitations to the current study that should be considered. First, FA may not be a true reflection of WM integrity, as it is possible that the altered structural connectivity exists but does not take the form of impaired structural integrity that could be detected by DTI. For example, tensor‐based tractography methods are especially sensitive to crossing fibers, where there are several different bundles of fibers within any given voxel that show different orientations. Although the final estimated FA value might reflect the predominant fiber orientation in any given voxel, the varying degrees of crossing fibers across different regions of the brain can still lead to biases in estimation, and hence inaccuracies in FA value [Tournier, Mori, & Leemans, 2011]. Therefore, it is important to note that the lack of FA differences may not necessarily assume any WM differences, as previous research has found axonal water fraction differences in the absence of FA differences in autism [Sui et al., 2018]. Interpretations of the findings should therefore be gauged with caution. Furthermore, the sample characteristics of the present study limit the generalizability of the findings.

Second, while the sample size exceeded some previous studies that employed similar methodology, our study sample remains moderate overall (ASD = 81; TD = 39). Examining females only, when taken into consideration that the current study completed group contrasts across diagnostic groups but within biological sex, the current sample size for females (ASD = 25, TD = 16) is slightly larger than that of Beacher et al. [2012] (ASD = 13, TD = 15), and that of Kirkovski et al. [2015] (ASD = 13, TD = 12), although is slightly smaller than that of Nordahl et al. [2015] (ASD = 27, TD = 29). Therefore, future studies should seek to explore WM differences in females with and without ASD by using much larger sample sizes comparable to the scale of studies conducted in males with ASD, to examine whether current findings can be replicated.

Third, the wide age range of the current study should also warrant future studies to evaluate the impact of lifespan development on sex differences. Given that previous studies investigating sex differences using DTI have included either children [Nordahl et al., 2015] or adults [Beacher et al., 2012; Kirkovski et al., 2015], the sex‐based differences attributable to development versus diagnosis need to be better disentangled. Future studies using a larger sample with a wider age range can investigate whether diagnosis may also lead to developmental differences in both sexes by conducting group comparisons of ASD versus TD within each sex. Additionally, the sample mostly included individuals with IQ above 70, and without any co‐occurring psychiatric or neurological co‐occurring conditions. Although the greater homogeneity in terms of both cognitive functioning and also lack of co‐occurring conditions in the current study may have limited potential noise introduced into the data when taken into consideration the relatively small to moderate sample sizes across the four groups, it also limits the generalizability of current study's results across the autism spectrum. Although it is challenging to collect DTI data from individuals with an IQ below 70, given that both intellectual disability and other co‐occurring conditions such as anxiety and depression can affect between 40% and 70% of children and young people with autism [White, Oswald, Ollendick, & Scahill, 2009], future studies should aim to replicate these findings in a larger sample size with a wider range of IQ scores, and examine how co‐occurring biomedical and psychiatric conditions might also influence potential WM differences observed through DTI.

Finally, using SRS‐2, the current study did not find any correlation between autism symptom severity/level of autistic traits and FA differences in females with and without ASD, although this might be due to broader concerns with many existing autism symptom measures not developed or are sensitive toward potential sex‐differences in phenotypic presentation of autism in females. Future studies should consider the female autism phenotype more carefully by employing assessment tools such as the Camouflaging Autistic Traits Questionnaire (CAT‐Q) [Hull et al., 2019]. Adding the CAT‐Q as an additional measure would provide a more sensitive measure of nuanced differences in autism symptom severity/level of autistic traits in social communication and help assess any potential relationships between neuroanatomical differences and differences in the level of camouflaging in females with and without ASD. Given that females with ASD may exhibit a more severely impaired behavioral phenotype, future neuroimaging studies should consider sex differences in the underlying neurobiology of ASD as these behaviors may be associated with identifiable, sex‐specific structural neural phenotype.

In conclusion, this study has demonstrated clear differences in brain connectivity in girls with ASD compared to TD girls, although were not seen in boys with and without ASD. Earlier studies without stringent data quality control should be interpreted with caution if robust data quality measures cannot be confirmed with confidence. Future studies should include larger samples and separate sex analyses, as well as more sensitive measures of autistic traits/symptom severity that can account for sex differences in autism behavioral presentation. Consideration should also be given to one‐to‐one matching on age and IQ for ASD and TD groups. The current findings thus further highlight that beyond considering phenotypic and neurobiological differences between autism and that of TD peers, some of these neurobiological differences could be sex‐specific, despite similar behavioral presentation of autism across both sexes. Gaining a better understanding of clear differences in neural phenotype would support the development of specific approaches unique to girls with autism, with implications for both diagnostic procedures and clinical interventions.

## Author Contributions

J.L. and R.J.J.: participated in the conceptualization, design, data collection, data analysis, data interpretation, and drafting and revising of this manuscript; E.L.: participated in data analysis, data interpretation, and drafting and revising of this manuscript; J.J., S.B., and K.A.P.: participated in the data interpretation, drafting, and revising of this manuscript; D.G.S. and P.V.: participated in the data collection, and revising of this manuscript.

## Conflict of Interest

All authors declare no conflict of interest for their contribution to the work carried out in this paper.
